# Effects of Formulation on the Palatability and Efficacy of In-Feed Praziquantel Medications for Marine Finfish Aquaculture

**DOI:** 10.3390/md20050323

**Published:** 2022-05-13

**Authors:** Edith K. Y. Tang, Gavin J. Partridge, Lindsey D. Woolley, Luke Pilmer, Lee Yong Lim

**Affiliations:** 1 School of Allied Health, Division of Pharmacy Department, University of Western Australia, Stirling Highway, Perth, WA 6009, Australia; edith.tang@uwa.edu.au; 2 Department of Primary Industries and Regional Development, Fleet Street, Fremantle, WA 6160, Australia; gavin.partridge@dpird.wa.gov.au (G.J.P.); lindsey.woolley@dpird.wa.gov.au (L.D.W.); luke.pilmer@dpird.wa.gov.au (L.P.); 3 Centre for Sustainable Aquatic Ecosystems, School of Veterinary & Life Sciences, Harry Butler Institute, Murdoch University, South Street, Murdoch, Perth, WA 6150, Australia; 4 Oceans Institute, University of Western Australia, Stirling Highway, Perth, WA 6009, Australia

**Keywords:** praziquantel, yellowtail kingfish, agar, chitosan, alginate, palatability, efficacy

## Abstract

Praziquantel (PZQ) provides an effective treatment against monogenean parasitic infestations in finfish. However, its use as an in-feed treatment is challenging due to palatability issues. In this study, five formulations of PZQ beads (1–4 mm) were developed using marine-based polymers, with allicin added as a flavouring agent. All formulations attained PZQ loading rates ≥74% *w*/*w*, and the beads were successfully incorporated into fish feed pellets at an active dietary inclusion level of 10 g/kg. When tested for palatability and digestibility in small yellowtail kingfish, the PZQ-loaded beads produced with alginate-chitosan, alginate-Cremophor^®^ RH40, and agar as carriers resulted in high consumption rates of 99–100% with no digesta or evidence of beads in the gastrointestinal tract (GIT) of fish fed with diets containing either formulation. Two formulations produced using chitosan-based carriers resulted in lower consumption rates of 68–75%, with undigested and partly digested beads found in the fish GIT 3 h post feeding. The PZQ-loaded alginate-chitosan and agar beads also showed good palatability in large (≥2 kg) yellowtail kingfish infected with gill parasites and were efficacious in removing the parasites from the fish, achieving >90% reduction in mean abundance relative to control fish (*p* < 0.001). The two effective formulations were stable upon storage at ambient temperature for up to 18 months, showing residual drug content >90% compared with baseline levels. Overall, the palatability, efficacy and stability data collected from this study suggest that these two PZQ particulate formulations have potential applications as in-feed anti-parasitic medications for the yellowtail kingfish farming industry.

## 1. Introduction

The economic cost of parasitism in cultured fish has been estimated at circa $US10 billion annually [1]. Monogenean ectoparasites are a highly diverse subset of this group that affect both marine and freshwater fish. Between 4000 and 5000 species have been described with many causing considerable economic impacts on fish farming industries globally [2,3]. Wild fish are commonly infected with monogeneans with little impact on their wellbeing; however, the parasites’ direct lifecycles and other ecological and reproductive features make confined (i.e., cultured) fish highly susceptible to uncharacteristically high levels of infection [3]. Left untreated, these parasites can cause growth impairment, anaemia, and secondary bacterial infections, leading to high morbidity and mortality [4]. A common treatment for infected fish in sea cages involves bathing in hydrogen peroxide [5,6], an expensive, labour-intensive, time-consuming, and weather-dependent method that can affect fish health [7] and pose significant occupational health risks for farm staff.

Praziquantel (PZQ) is a highly efficacious anthelmintic agent [8,9] against a wide range of platyhelminth parasites, including monogeneans. It is effective when administered to infected fish via injection, bathing, or feeding [4,10,11,12,13,14,15,16,17]. Injection, however, is impractical in commercially farmed fish, and bathing presents challenges similar to those described above for hydrogen peroxide, with the added disadvantage of discharging relatively large quantities of a pharmacologically active agent into the environment. Feeding can potentially deliver PZQ into a large number of fish in a stress-free manner; however, the bitter taste of PZQ is a major constraint to effective delivery [4,11,12,15,18,19]. Significant formulation research has been conducted on masking the bitterness of PZQ to improve its palatability but with little success, particularly in *Seriola* species. This group represents the fourth most valuable cultured marine fish industry in the world [20], predominantly from Japan, and with rapid expansion into new regions, including Australia, New Zealand, South Africa, Europe, and the Americas [21]. In these regions, the various *Seriola* species are infected with monogenean parasites (*Benedenia seriolae*, *Zeuxapta seriolae*, and *Neobenedenia melleni*), the management of which contributes up to 20% of the cost of production [22,23,24], at a cost of circa $0.5 billion in Japan alone [1].

The palatability issues of PZQ in *Seriola* have been well described [4,18,19]. In an effort to address this issue, Partridge et al. (2014) tested microencapsulated PZQ, which improved palatability, but reduced bioavailability compared with pure PZQ [4]. Incorporation of PZQ into solid lipid nanoparticles with and without a chitosan coating also failed to achieve acceptable palatability and bioavailability [11]. Similarly, delivery of only the R-(−) enantiomer of PZQ in feed failed to improve its palatability relative to racemic PZQ or the S-(+) enantiomer [12]. Pilmer (2016) evaluated various taste masking agents with only limited improvements to palatability [25]. In 2016, Forwood et al. effectively masked the flavour of PZQ to yellowtail kingfish using moist pellets; however, these have the disadvantage of having to be prepared fresh (at sea) just prior to use [26].

This study aimed to develop effective taste masked particulate PZQ formulations capable of being incorporated into fish feed to provide in-feed anti-parasitic medications for cultured yellowtail kingfish (*Seriola lalandi*). The beads were formulated to control PZQ availability by withholding drug release in seawater to minimise detection, thus encouraging feeding by the fish, and once consumed by the fish, were digestible in the gastrointestinal tract (GIT) to provide a bolus drug dose. Beads were fabricated using sodium alginate, agar, and chitosan, applied individually and in combinations, as carriers. The polymers are generally regarded as safe (GRAS) materials derived from marine sources [27], and have been used as vehicles for drug delivery [28,29,30,31,32,33,34,35] and binders for fish feed [36,37,38]. Garlic derivatives, including allicin, were applied as flavours based on literature evidence [25].

## 2. Results

### 2.1. Bead Characterisation

#### 2.1.1. Bead Size and Drug Loading

All formulations achieved a drug loading of at least 74% *w*/*w* PZQ in the dry beads (Table 1). Increasing the batch size by 10-fold did not affect the efficiency of drug loading in Formulations B, C, and D (Table 1). The formulations had mean dry bead diameters ranging from 1.36 to 3.03 mm (Table 1). Formulation B had the largest bead diameter due to the incorporation of insoluble chitosan particles in the alginate matrix for these beads.

#### 2.1.2. In Vitro Disintegration Profile

**Figure 1:**
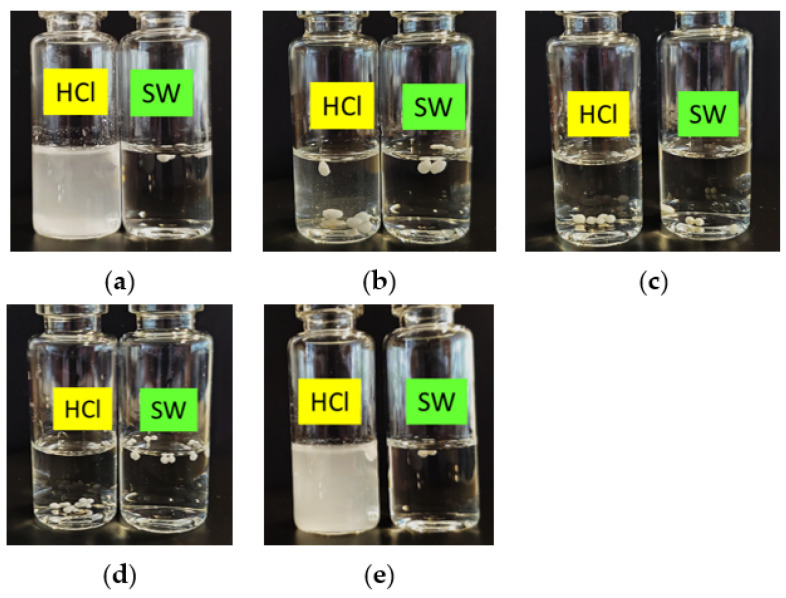
In vitro evaluation of the disintegration of (**a**) Formulation A, (**b**) Formulation B, (**c**) Formulation C, (**d**) Formulation D, and (**e**) Formulation E after 5 h incubation at ambient temperature in seawater (SW) and simulated gastric fluid of fish (0.1 M HCl).

Qualitative results of the bead disintegration in seawater and 0.1 M HCl are shown in Figure 1. As shown in the figure, none of the beads from the five formulations disintegrated in seawater, as the medium remained clear and intact beads were still present after 5 h of incubation. Beads from Formulations A and E, prepared with chitosan only as carrier, showed complete disintegration in 0.1 M HCl to yield white suspensions with bead fragments. Formulation B turned the 0.1 M HCl solution slightly turbid after 5 h, suggesting evidence of bead disintegration; however, the disintegration was incomplete as the beads did not show any breakdown of structure (Figure 1b). No disintegration of the beads was observed for Formulations C and D in 0.1 M HCl.

#### 2.1.3. In Vitro Dissolution Profile of Formulation B and Formulation C

**Figure 2:**
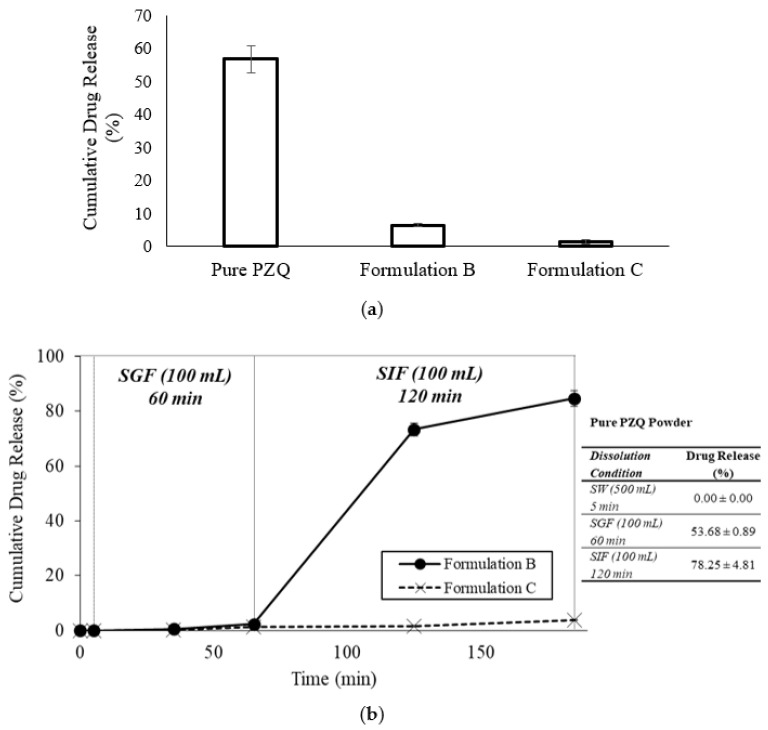
(**a**) Cumulative percent dissolution of praziquantel (PZQ) from pure drug powder, Formulation B, and Formulation C after 180 min incubation in seawater. (**b**) PZQ release profiles for Formulation B and Formulation C beads incubated sequentially in different media: SW = seawater, SGF = simulated gastric fluid, SIF = simulated intestinal fluid. Table shows cumulative PZQ release from pure drug powder under specified dissolution conditions. Data represent mean ± SD (*n* = 3).

Dissolution profile of PZQ for Formulation B and Formulation C were studied by simulating the passage of the beads from seawater into the fish GIT. This simulation could not be performed for the pure drug powder using the basket apparatus; instead, the dissolution profile of the pure drug was determined separately in each medium. Dissolution of PZQ was not detectable after 5 min in seawater; however, 56.7 ± 4.1% of the drug powder had dissolved after 3 h in 500 mL of seawater (Figure 2a), which was comparable to the 53.7 ± 0.9% dissolution obtained after 1 h in 100 mL of SGF (Figure 2b). Incubation for 2 h in SIF led to the dissolution of 78.2 ± 4.8% of the drug powder (Figure 2b). There was incomplete dissolution of the PZQ powder under the simulated GIT conditions, although the drug:media ratio (7–13 mg in 100 mL) was below the solubility of PZQ in water (approximately 36 mg/100 mL) [39].

Formulation B and Formulation C beads did not disintegrate even after 3 h incubation in 500 mL of seawater, and only 6.51 ± 0.59% and 1.36 ± 0.71% of the drug loads from the respective beads were leached into the seawater at 3 h (Figure 2a). Simulation of the bead passage from seawater into the fish GIT showed undetectable drug release after 5 min in seawater from both formulations. The Formulation B beads remained intact after a further 60 min incubation in SGF, releasing only 2.3 ± 0.4% of the drug load; however, bead disintegration was noted in the SIF accompanied by the release of 84.7 ± 2.9% of the drug load at 185 min (Figure 2b). In contrast, the beads of Formulation C remained intact not only in seawater, but also in the SGF and SIF, with low levels of drug release measured over the entire dissolution period. Cumulative percent drug release from the Formulation C beads was only 3.87 ± 0.24% at 185 min.

#### 2.1.4. PZQ Compatibility with Matrix Materials in Formulations B and C

**Figure 3:**
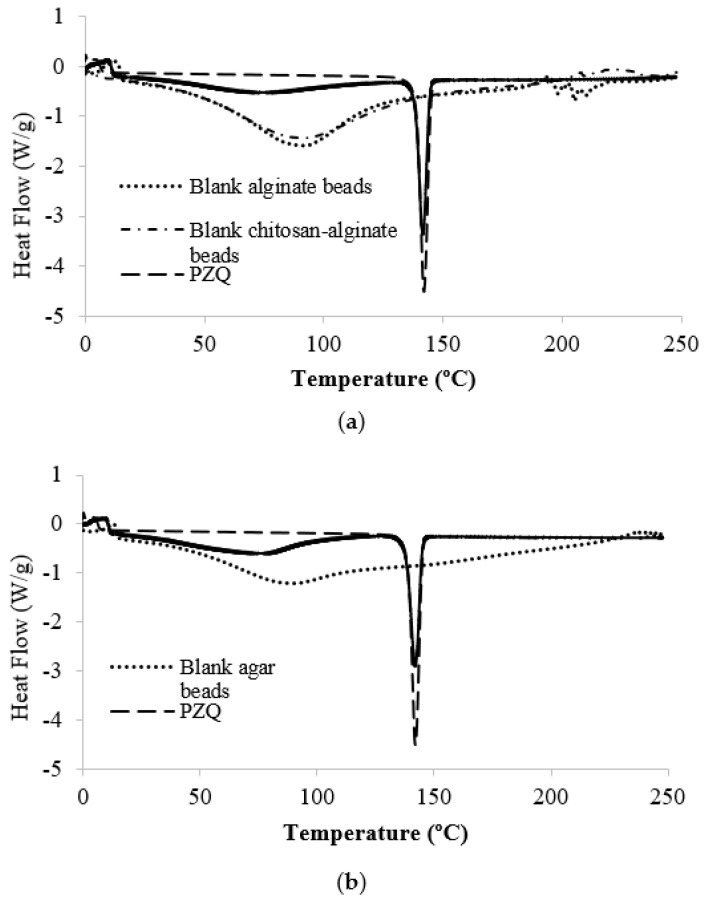
DSC thermograms of (**a**) Formulation B and (**b**) Formulation C in comparison with the DSC thermograms of PZQ powder and the corresponding blank beads.

DSC analysis was conducted for Formulations B and C, and the thermograms were compared with the DSC thermograms of PZQ and the corresponding blank beads in Figure 3. PZQ (3.4 mg) exhibited a sharp melting endotherm with onset at 138.9 °C and peak temperature of 141.99 °C. The enthalpy for melting was 99.870 J/g. PZQ melting peak for Formulation B (3.8 mg) had onset at 138.35 °C and peak temperature at 141.50 °C. Its enthalpy of 72.607 J/g was 72.70% that of pure PZQ, and corresponded to the drug loading of Formulation B. PZQ peak in Formulation C (3.4 mg) had onset at 138.03 °C and peak temperature at 141.93 °C. Its enthalpy of 71.645 J/g was 71.74% that of pure PZQ, which again corresponded closely to the drug loading of Formulation C. Thus, it may be concluded from the respective DSC thermograms that PZQ retained its crystalline characteristics, and did not interact with the matrix materials in Formulations B and C.

### 2.2. Storage Stability of Formulations B and C BEADS at Ambient Temperature

Beads of Formulations B and C were stored in sealed plastic containers at ambient temperature, protected from light for 18 months. Their residual PZQ content was observed to be 90–110% of baseline PZQ content (measured immediately after manufacture), suggesting that both formulations are stable when stored for at least 18 months.

### 2.3. Palatability and Digestibility of Formulations A, B, C, D, and E in Healthy (Uninfected) Fish

**Figure 4:**
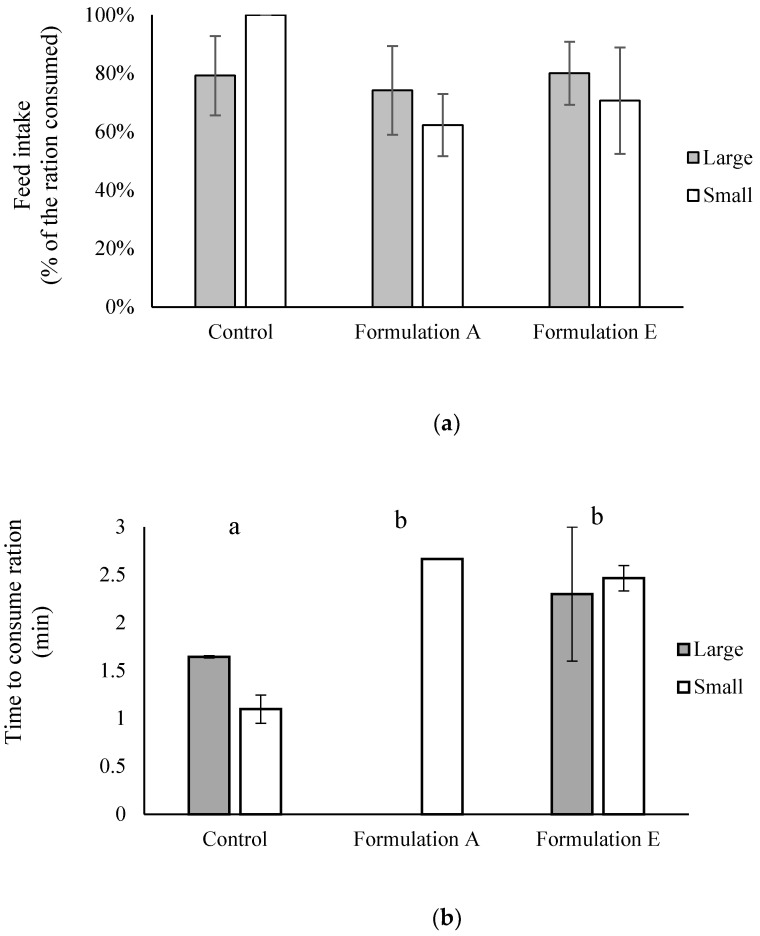
Palatability evaluation of fish feed incorporated with Formulation A or Formulation E fed to large (2000 g, *n* = 2 fish per tank) and small (175 g, *n* = 8 fish per tank) yellowtail kingfish. A fixed ration of 72 g and 48 g of food per tank per day was offered in a single morning feed for small and large fish, respectively. (**a**) Overall consumption of diet ration and (**b**) time taken by fish to consume the entire ration. Different lowercase letters indicate significant differences between treatments. Data are presented as mean ± SE with day of treatment as replicate (*n* = 5).

Analysis by two-way ANOVA showed no effect of fish size (*p =* 0.77) or treatment (*p =* 0.07) on overall consumption of the medicated feeds incorporating Formulations A and E, both of which contained chitosan only in the bead matrix. Large fish fed the unmedicated control treatment consumed 79 ± 14% of the ration, while those fed Formulation A ate on average 74 ± 15%, and fish fed Formulation E ate 80 ± 11% of the ration (Figure 4a). The small fish fed the unmedicated control diet ate their entire ration, but only ate 62 ± 11% and 71 ± 18% of Formulation A and Formulation E treatment rations, respectively. There was no significant effect of fish size on the time taken to consume the fixed ration (*p =* 0.212); however, there was a significant difference between the treatments (*p <* 0.01; Figure 4b). On those occasions when the fish ate the entire medicated ration, the time to do so was circa 3 min. Fish offered the unmedicated control treatment consumed their ration in circa 1.5 min. The large yellowtail kingfish fed with Formulation A did not eat an entire ration of feed during the 5-day trial and the small fish only ate the full ration of Formulation A on one occasion.

**Figure 5:**
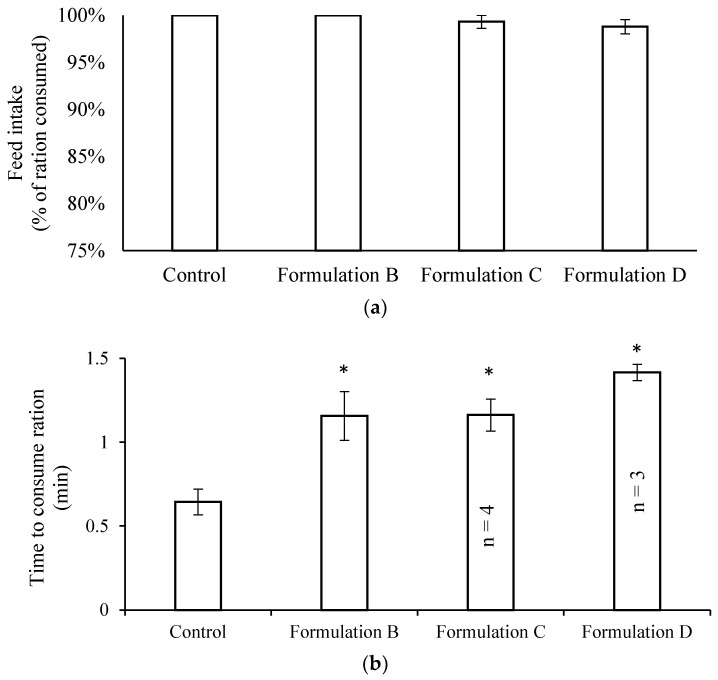
Palatability evaluation of fish feed incorporated with Formulation B, Formulation C or Formulation D fed to small yellowtail kingfish (260 g, *n* = 10 per tank). Fish were offered a fixed ration of 78 g of food per tank per day in a single morning feed. (**a**) Overall consumption of diet ration and (**b**) time taken by fish to consume the entire ration. Data are presented as mean ± SE with day of treatment as replicate (*n* = 5 unless indicated on figure). * Significant difference compared to unmedicated control.

As there was no evidence of fish size affecting PZQ palatability, only small fish were subsequently used for assessing the palatability of Formulations B, C, and D. The fish ate ≥99% of all treatments (Figure 5a), with no significant difference between treatments (*p =* 0.28, one-way ANOVA). However, whilst the fish ate their full ration at each feeding, the time taken to consume the ration was significantly different between all formulations and the unmedicated control (0.64 ± 0.07 min) (*p <* 0.02). The times taken to eat a complete ration of diets containing Formulation B and Formulation C were 1.15 ± 0.14 min and 1.16 ± 0.14 min (*n* = 4), respectively, while Formulation D took the longest at 1.42 ± 0.04 min (*n* = 3) (Figure 5b). On the basis of the slower time taken to consume the diet containing Formulation D, it was not progressed to the efficacy trials.

**Figure 6:**
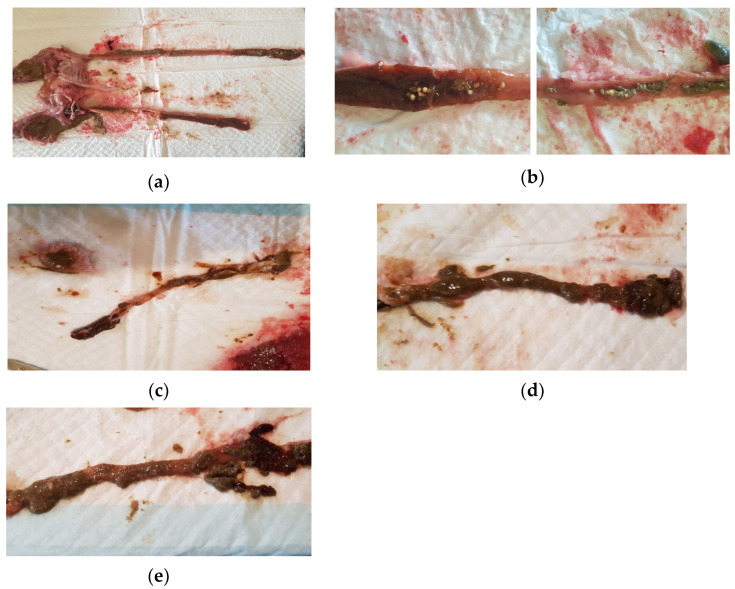
Gastrointestinal tract of yellowtail kingfish dissected 3 h post feeding of medicated in-feed. (**a**) No evidence of undigested beads in the intestinal tract of large yellowtail kingfish fed Formulation A; (**b**) Intestinal tract of a large yellowtail kingfish fed Formulation E showing undigested beads in the (1) midgut and (2) hindgut; (**c**) Intestinal tract showing no evidence of beads for small yellowtail kingfish fed Formulation B, (**d**) Formulation C, and (**e**) Formulation D.

With regards to digestibility, there was no evidence of beads found in the digestive tract in either small or large fish fed the Formulation A treatment (Figure 6a). However, undigested and partly digested beads of the Formulation E treatment were found throughout the entire length of the intestinal tract in the large fish (Figure 6b). For the other three treatments, apart from a single Formulation D bead in the stomach, there was no digesta or evidence of beads in the intestinal tracts of fish fed with diets containing Formulations B, C, or D (Figure 6c–e).

### 2.4. Palatability and Efficacy of Formulations B and C in Parasite-Infected Fish

**Figure 7:**
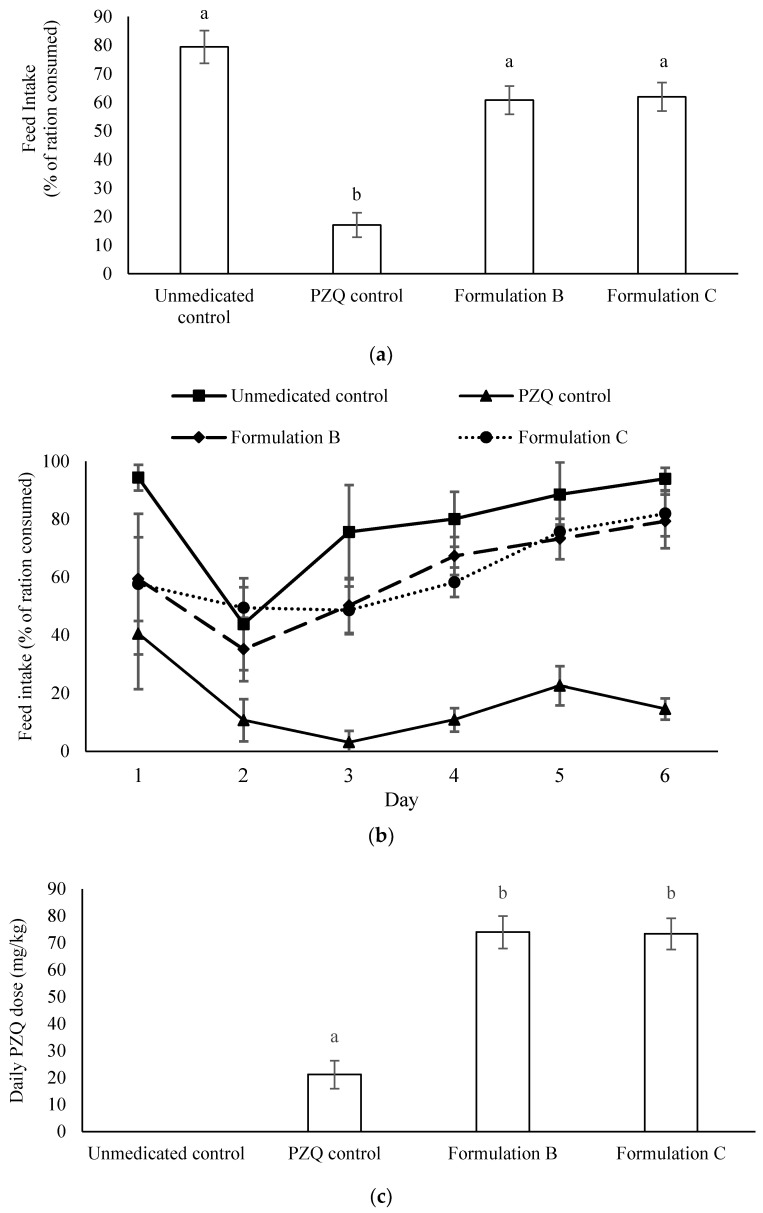
Feed intake and praziquantel (PZQ) dose received by yellowtail kingfish infected with Zeuxapta parasites and provided with fish feed containing no PZQ (unmedicated control), PZQ powder (PZQ control), and Formulations B and C. Twelve tanks, each stocked with eight fish (1600 g), were used to evaluate the treatments in triplicate. Fish were offered rations of 155 g of fish feed per tank per day in a single morning feed over 6 days. (**a**) Percentage of feed ration consumed, (**b**) daily ration intake, and (**c**) daily PZQ dose (mg/kg) received by the fish. Lowercase letters indicate significant differences between treatments. Data presented as mean ± SE, *n* = 3.

For the palatability and efficacy trial in parasite infected fish, the fish ate significantly less of the positive PZQ (powder) control treatment (17 ± 4%) than that of the unmedicated control treatment (79 ± 6%) (*p <* 0.001, Figure 7a). However, there was no significant difference between the intake of fish fed the unmedicated control and those fed with Formulation B (61 ± 5%) or Formulation C (62 ± 5%). Figure 7b shows the feed intake by day, demonstrating that the fish in all the tanks fed poorly on the second day, and that the feed intake subsequently increased for all treatment groups over the following days, with the exception of the PZQ control treatment group, which consistently showed low feed intakes. The intake of diets containing Formulation B and Formulation C increased with time, mirroring the trend of the unmedicated control group.

Figure 7c presents the average daily drug dose, calculated as mg PZQ per kg body weight based on ingested feed received by the fish in the different treatment groups. The mean daily PZQ dose ingested by fish fed the PZQ control treatment was 21 ± 5 mg/kg, which was significantly lower than the PZQ doses received by fish fed with the Formulation B (74 ± 6 mg/kg) and Formulation C (73 ± 6 mg/kg) treatments (*p <* 0.001).

**Figure 8:**
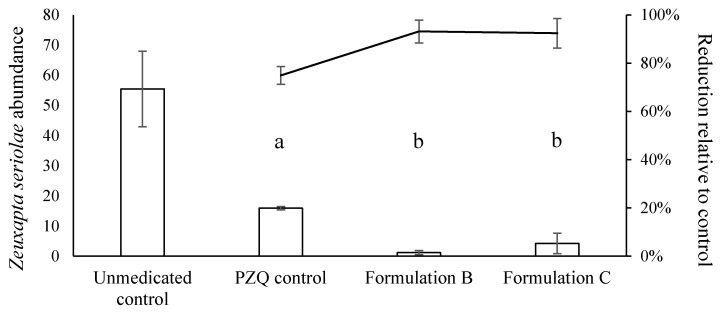
Mean abundance of Zeuxapta parasites infecting yellowtail kingfish after 6 days of treatment with unmedicated feed, and feed containing praziquantel powder (PZQ control), Formulation B, and Formulation C, compared against the percent reduction relative to the unmedicated feed. Different letters indicate significant differences between treatments. Data presented as mean ± SE, *n* = 3.

At the end of the 6-day trial period, fish fed the unmedicated control diet had a mean abundance of 55 ± 13 Zeuxapta gill parasites per fish. There was a significant reduction in parasite abundance in the three PZQ treatment groups relative to this unmedicated control (*p <* 0.001; Figure 8). Fish fed diets containing Formulation B and Formulation C had parasite reductions of 93 ± 2% and 94 ± 3%, respectively, both higher than that seen in the positive PZQ control, where a 73 ± 4% reduction in parasite abundance was observed. The percentage reduction in parasites relative to the negative control was significantly higher in fish fed Formulation B and Formulation C than the positive control (*p <* 0.001). Gut dissections showed no whole beads retained in either the stomach or gut of the fish. However, there was evidence of white mucous, and it is not known whether this was from the PZQ beads or just a normal digestive function.

## 3. Discussion

Though widely regarded to be an efficacious drug for the treatment of fish infected with monogenean parasites, praziquantel (PZQ) is currently constrained in its use as an in-feed treatment because of its bitter taste. In this study, we successfully developed two PZQ particulate formulations using a combination of alginate and chitosan (Formulation B) and agar alone (Formulation C) as the bead matrix, both also containing allicin powder as a flavouring agent. The beads of Formulations B and C were shown not to disintegrate in seawater and could be incorporated into fish feed pellets with high palatability for small and large yellowtail kingfish. Unlike the preparation of praziquantel nanoparticles using chitosan-N-arginine and alginate [40,41], the manufacturing processes for these beads are less complex and can easily be up-scaled, and the beads are stable to storage at ambient temperature. More importantly, they were demonstrated to be efficacious against gill parasites, indicating that the incorporated PZQ was released from the beads in vivo for bioactivity in the fish. Prior research has demonstrated that microencapsulating PZQ can improve its palatability to yellowtail kingfish [4]; however, these microcapsules were damaged by the heat and/or pressure of the extrusion process used to manufacture the fish diets, resulting in leaching of PZQ into the medicated feed and a subsequent reduction in palatability. Surface coating fish feed post-extrusion with solid lipid nanoparticles containing PZQ has also been trialled unsuccessfully to improve PZQ palatability to yellowtail kingfish. A major advantage of the beads produced in the current study was the high loading rate (>74%) compared to the aforementioned microcapsules (40%) [4] and solid lipid nanoparticles (<10%) [11]. Such low loading rates necessitate much higher inclusion levels of these particles to achieve the same active dietary inclusion level of PZQ and was hypothesised to contribute to the lack of palatability improvement.

In this study, alginate, chitosan, and agar were used to formulate the bead matrix as they are extracted from marine sources [27], and alginate and agar are commonly used as binders for fish feed [36,37]. Preliminary formulations that used alginate alone as the bead matrix (i.e., without chitosan) resulted in improved palatability of PZQ compared to unencapsulated PZQ; however, the alginate beads were not digestible in vivo, and intact beads were found along the length of the dissected GIT of treated fish (data not shown). The poor digestibility was attributed to the poor solubility of alginate and PZQ in the acidic conditions of the fish stomach [42]. As the polycationic chitosan dissolves under acidic conditions [43] and chitin is known to be digestible by fish [44], chitosan alone was used to prepare the PZQ-loaded beads in Formulations A and E. The chitosan beads showed better digestibility in the fish gut; however, palatability was reduced, which could be attributed to PZQ being released from the beads into seawater. Prolonging the complexation time between chitosan and the tripolyphosphate (TPP) ions from 3 h (Formulation A) to overnight (Formulation E) did not resolve the palatability; instead, it reduced the bead digestibility in vivo. It was concluded that the acidic solvent used to dissolve the chitosan polymer might have a residual acidic (sour) taste, or the premature disintegration of the chitosan beads prior to reaching the fish stomach might have adversely affected palatability.

To make the palatable PZQ-loaded alginate beads more digestible in the fish gut, chitosan and Cremophor^®^ RH40 were trialled as additives. Microparticles having a mix of alginate and chitosan have been fabricated by several methods, including co-dissolving sodium alginate and chitosan in an acidic solvent and extruding this solution into a calcium chloride solution [45]; extruding an aqueous solution of sodium alginate into a solution containing chitosan and calcium chloride dissolved in an acidic solvent [28]; incubating freshly formed calcium alginate beads in a chitosan solution [33]; or extruding a chitosan solution into a TPP solution containing sodium alginate [34]. Beads fabricated using these methods aim to produce an external layer of crosslinked alginate-chitosan, which is not favoured for PZQ as the crosslinked polymer would adversely affect digestibility in vivo. Instead, the PZQ-loaded alginate-chitosan beads (Formulation B) were prepared by mixing a powder comprising PZQ, allicin, and chitosan into the alginate solution and extruding the suspension into a calcium chloride solution. The chitosan powder served as a wicking agent, which upon dissolution in the acidic gastric fluid in the fish would increase porosity and capillary action in the beads to enhance bead digestibility and PZQ release. Conversely, Cremophor^®^ RH40 (Formulation D) was used as a surfactant solubiliser, allowing not only smaller beads to be made, but also enhancing the wettability and digestibility of the beads in vivo. Formulations B and D did not disintegrate in seawater, nor did they disintegrate completely in 0.1 M HCl; however, the initially floating beads were observed to sink after 5 h of incubation in the acidic medium, indicating water intake. Formulation B showed some evidence of disintegration, turning the medium slightly turbid, and the in vitro dissolution study suggested that Formulation B beads disintegrated to allow PZQ release in the fish GIT. Both formulations, when incorporated into fish feed, were palatable and digestible for the yellowtail kingfish. Formulation D was less palatable than Formulation B, based on the time taken for the fish to completely eat the given feed ration, and this is attributed to Cremophor^®^ RH40 being a surfactant; it could have facilitated the dissolution and leaching of PZQ from the beads into the seawater, leading to the detection of the drug by the fish.

Agar used in Formulation C was digestible by the yellowtail kingfish, which is not surprising, as it is a marine product that is also widely used as a binder for fish food [37]. Agar has been used to provide sustained drug release [35], but it is not as widely used as alginate and chitosan for drug delivery, possibly because heat is required for its manufacture, making it inaccessible to heat labile drugs. PZQ is, however, very heat stable [46]. In this study, the PZQ-loaded agar beads (Formulation C) showed similar palatability, in vivo digestibility, and efficacy to Formulation B. Unlike Formulation B, the Formulation C beads did not disintegrate in seawater, SGF, or SIF, and the in vitro dissolution data showing low levels of PZQ release were not reflective of the in vivo digestibility and efficacy data. The dissolution media were adjusted for pH, surface activity, and enzymes (pepsin and trypsin) to represent the fish gastrointestinal fluids; however, the compositional content of the fish gut is not known with certainty, and it may well be that in vivo, the Formulation C beads were digested in the fish GIT by bacteria or other enzymes.

In the efficacy trial, fish in the PZQ control treatment group received an average PZQ dose over the 6 days of 21 mg/kg, which was adequate to provide a 73% reduction in parasite abundance. This appears to be in contrast to Forwood et al. [26] who found no reduction in gill parasites when yellowtail kingfish were intubated daily for 3 days with moist pellets containing 30 mg/kg of PZQ. The apparent discrepancy can be explained by the decreasing intake of the PZQ control diet over time i.e., whilst the average dose over 6 days was 21 mg/kg, the fish ate a much higher percentage of their ration on day 1, equivalent to a dose of 49 mg/kg. It is, therefore, likely that most of the parasites in this treatment were dislodged on the first day.

The average daily dose of PZQ received by fish in the Formulation B and Formulation C treatment groups was more than three times higher than those in the positive control treatment. This dose yielded a 93% and 94% reduction in gill parasites, respectively. Whilst Forwood et al. demonstrated that it is possible for a single dose of 50 mg/kg to eliminate >97% of gill parasites, in a commercial context, medicated feeds are offered over a longer period of time (3–7 days) to overcome the variation in intake between individual fish on a day-to-day basis [26]. Complete parasite elimination was not achieved with either formulation since not all fish fed equally. Indeed, Forwood et al. highlighted that competitive feeding is much stronger in a larger (commercial) population of fish, which may facilitate a more uniform intake [26].

An added advantage is that Formulation B and Formulation C are stable to store at ambient temperature for up to 18 months, while the storage stability of other formulations is not often reported. One final attraction of Formulation B for fish farmers is that chitosan as a supplement for fish feed has been found to reduce mortality and improve the growth performance of cultured marine fish [47].

## 4. Materials and Methods

### 4.1. Materials

Praziquantel (PZQ) was from TNN Development Limited (Dalian, China). Agar powder (Swallow Globe International, Casula, NSW, Australia), soybean oil (Sun Horse, VHT, Morley, WA, Australia), and cod liver oil (Gold Cross, Barton, ACT, Australia) were purchased from local stores. Sodium alginate (low viscosity grade) was from BÜCHI Labortechnik (Flawil, Switzerland), while trypsin, pepsin, chitosan (medium molecular weight), and sodium tripolyphosphate (TPP) were from Sigma Aldrich (Sydney, NSW, Australia). Calcium chloride, hydrochloric acid, and glacial acetic acid were purchased from ChemSupply (Gillman, SA, Australia). Sodium chloride, potassium chloride, sodium hydrogen phosphate, and potassium dihydrogen phosphate were from Ajax Chemicals (Sydney, NSW, Australia). Allicin powder (25%, calcium carbonate base) and allicin oil were from Hebei Kangdali Pharmaceutical Co., Ltd. (Hebei, China) and hydrogenated polyoxyl 40 castor oil (Cremophor^®^ RH40, BP grade) was from Ingredient Plus (Rydalmere, NSW, Australia). Polysorbate 80 NF was purchased from PCCA (Houston, TX, USA). HPLC grade methanol and acetonitrile were purchased from Merck (Darmstadt, Germany) and double deionised water (PSI Water Filters, South Launceston, TAS, Australia) was used throughout. Seawater (salinity 35 ppt) was obtained from a bore at a marine fish hatchery (Department of Primary Industries and Regional Development (DPIRD), Fremantle, Australia).

### 4.2. Bead Preparation

#### 4.2.1. Chitosan-Based Beads (Formulation A and Formulation E)

For a 10-g batch, PZQ (9 g) and allicin powder (0.5 g) were mixed into 100 mL of 1% *w*/*v* chitosan dissolved in 0.2 M acetic acid. The suspension was transferred into a 5 mL plastic syringe and extruded dropwise through a blunt needle (gauge 21) into 500 mL of 2% *w*/*v* aqueous TPP solution at ambient temperature. Beads were hardened in the TPP solution for 3 h (Formulation A) or overnight (>10 h) (Formulation E), washed thrice with water, and air dried over seven days. The dry beads were mixed with allicin powder (1% of dry bead weight) by shaking in a sealed plastic container and stored at ambient temperature until use.

#### 4.2.2. Alginate/Chitosan-Based Beads (Formulation B and Formulation D)

To prepare 10-g batches, PZQ (9 g), chitosan (1 g) and allicin powder (0.5 g) were mixed to prepare a powder for Formulation B, and PZQ (9 g), Cremophor^®^ RH40 (1 g), and allicin powder (0.5 g) were mixed to prepare a powder for Formulation D. Each powder mix was stirred into 100 mL of 1% *w*/*v* aqueous sodium alginate solution with sonication for 10 min (Ultrasonics, NSW, Australia), and the resultant suspension extruded through a 3 mL plastic transfer pipette with modified tip (Sarstedt Australia, Mawson Lakes, SA, Australia) into 200 mL of 1% *w*/*v* aqueous calcium chloride solution in a 250 mL measuring cylinder. The beads were hardened in the calcium chloride solution over 15 min at ambient temperature, washed thrice with water, and air dried over seven days. Once dry, the beads were mixed with allicin powder (1% of dry bead weight) by shaking in a sealed plastic container and stored at ambient temperature until use.

#### 4.2.3. Agar-Based Beads (Formulation C)

To prepare a 10-g batch, a powder mix of PZQ (9 g) and allicin powder (0.5 g) was stirred into 100 mL of a 2% *w*/*v* agar solution, prepared by dissolving agar in hot water. The resultant suspension, maintained at 60–80 °C, was transferred into a 1 mL glass syringe and extruded dropwise through a blunt needle (gauge 21) into 400 mL of a cold oil comprising allicin oil, soybean oil, and cod liver oil (5:3:1 *v*/*v*), in a 600 mL beaker placed on ice. The agar beads were hardened in the oil for 30 min, washed thrice with water at ambient temperature and air dried for seven days. The dry beads were mixed with allicin powder (1% of dry bead weight) by shaking in a sealed plastic container and stored at ambient temperature until use.

#### 4.2.4. Scaled-Up Manufacture

Formulations B, C, and D were also prepared as 100-g batches. The scaled-up batches were prepared using methods similar to those described for the respective 10-g batches; however, the ingredients in each formulation were increased 10-fold.

### 4.3. Bead Characterisation

#### 4.3.1. Bead Diameter

Twenty dry beads were randomly selected from each formulation and their diameters measured using a vernier calliper (Absolute Digital Digimatic, Mitutoyo, Aurora, IL, USA). Due to the oval shape of beads in Formulation B, the shortest and longest lengths of each bead were measured, and the mean value of the two lengths calculated as the bead diameter.

#### 4.3.2. Drug Loading and Stability on Storage

PZQ content in the beads was determined using the high-performance liquid chromatography (HPLC) assay described in the British Pharmacopoeia [48]. Baseline drug content was measured on day of manufacture (Day 0) and residual drug content determined after every three months of storage for 18 months at ambient temperature, protected from light. Triplicate samples of dry beads (approximately 20 mg) were weighed into 100 mL volumetric flasks and disintegrated in 25 mL of 0.4 M NaCl with sonication at 80–100 °C. Methanol was added with sonication for 5 min to dissolve the PZQ, and the solution upon cooling was made to volume with methanol. HPLC assay was conducted on an Agilent system (Agilent 1260 Infinity, Agilent Technologies Australia, Mulgrave, VIC, Australia) equipped with a Hypersil ODS C18 column (250 × 4.6 mm, 5 µm) (Thermo Fisher Scientific, Waltham, MA, USA). Isocratic elution with acetonitrile and water (1:1 *v*/*v*) at flow rate of 1.0 mL/min was employed, and PZQ was detected at 210 nm and 250 nm. The HPLC was calibrated with standard solutions of PZQ (4 to 400 µg/mL) dissolved in 25:75 *v*/*v* of 0.4 M NaCl:methanol. Sample and standard solutions, after filtration (0.22 µm, Phenomenex, Lane Cove West, NSW, Australia) into amber vials (Agilent Technologies Australia, Mulgrave, VIC, Australia), were injected at 10 µL for HPLC analysis. Drug loading was calculated as the amount of PZQ per unit dry bead weight.

#### 4.3.3. In Vitro Disintegration Profile

Approximately five dry beads from each formulation were placed into 20 mL glass vials containing 10 mL of seawater or 0.1 M hydrochloric acid (HCl) stirring at 100 rpm on a magnetic stirrer (IEC Equipment, Thornbury, Australia) at ambient temperature. Bead morphology in the medium was monitored visually over 5 h.

#### 4.3.4. In Vitro Dissolution Profile of Formulation B and Formulation C

**Figure 9:**
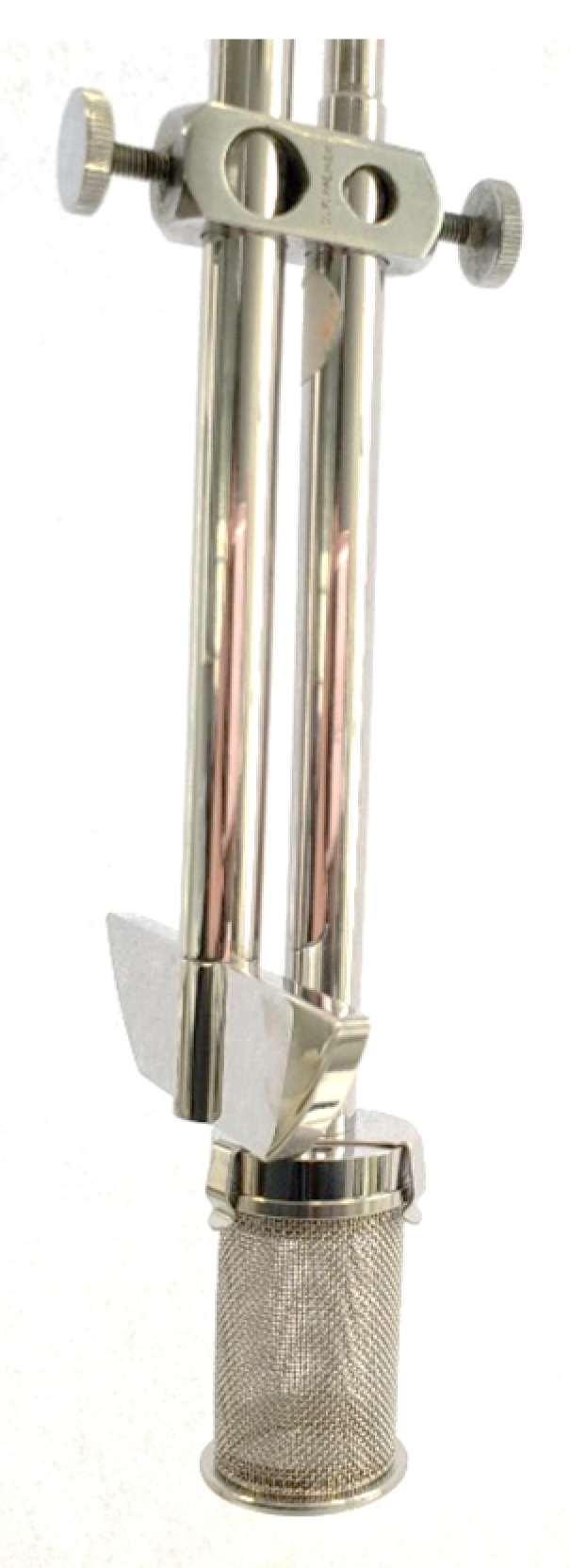
Dissolution apparatus set-up. Attachment of a basket holding the praziquantel-loaded beads to a paddle shaft to allow the beads to be submerged into 100 mL of dissolution medium, effective transfer of beads from one medium to the next, and stirring of the media during the dissolution experiments.

Approximately 10 mg of dry beads of Formulation B and Formulation C were separately weighed and transferred to USP dissolution baskets (708-DS model, Agilent Technologies, Mulgrave, VIC, Australia), each of which was then attached to a USP dissolution paddle shaft (Figure 9). The set-up ensured the beads were able to be submerged in a dissolution medium of low volume; beads were not removed during media sampling, medium could be agitated during dissolution, and beads could be quickly and collectively transferred from one medium to the next. Dissolution profiles were performed in triplicate at stirring speed of 100 rpm and ambient temperature (22–25 °C). The beads were incubated for 5 min in 500 mL of seawater, followed by 60 min in 100 mL of simulated fish gastric fluid (SGF, seawater adjusted to pH 2.0 ± 0.05 with HCl, with 0.1% *w*/*v* Polysorbate 80 and 0.8 mg/mL pepsin) and finally 120 min in 100 mL of simulated fish intestinal fluid (SIF, consisting of PBS 137 mM NaCl, 2.7 mM KCl, 10 mM Na2HPO4, and 1.8 mM KH2PO4, pH 7.8 ± 0.05 with 0.1% *w*/*v* Polysorbate 80 and 0.4 mg/mL trypsin) [49,50,51,52]. The respective dissolution medium was sampled (1 mL) at 0 and 5 min (seawater), 35 and 65 min (SGF), and 125 and 185 min (SIF). Withdrawn samples were filtered (0.45 μm) and analysed for praziquantel content using the HPLC assay. Control experiments were performed using pure PZQ powder (7–13 mg per basket). However, as the basket could not be transferred from one dissolution medium to the next without loss of powder, the experiments for the PZQ powder were performed separately in the three dissolution media as follows: 5 min in 500 mL of seawater, 60 min in 100 mL of SGF, and 120 min in 100 mL of SIF. Additionally, the dissolution profiles of Formulation B and Formulation C beads were determined over 3 h in seawater using the same equipment set-up.

#### 4.3.5. Analysis by Differential Scanning Calorimetry (DSC)

Formulation B, Formulation C, blank agar, alginate and alginate-chitosan beads, and PZQ alone were analysed in a differential scanning calorimeter (Discovery DSC25 System, TA Instruments, Newcastle, DE, USA). Bead samples were analysed within 14 days of manufacture. Samples (~3 mg) were analysed in standard aluminium pans (DSC Consumables Incorporated, Austin, MN, USA) over 0 to 250 °C at a heating rate of 10 °C/min with empty aluminium pans as reference. DSC thermograms were analysed using the TRIOS Software (TA Instruments, New Castle, DE, USA).

### 4.4. Palatability Assessment in Healthy Fish

Palatability and efficacy trials were conducted at DPIRD’s Marine Fish Facilities with approval from DPIRD animal ethics committee (permit number 20-5-15).

**Figure 10:**
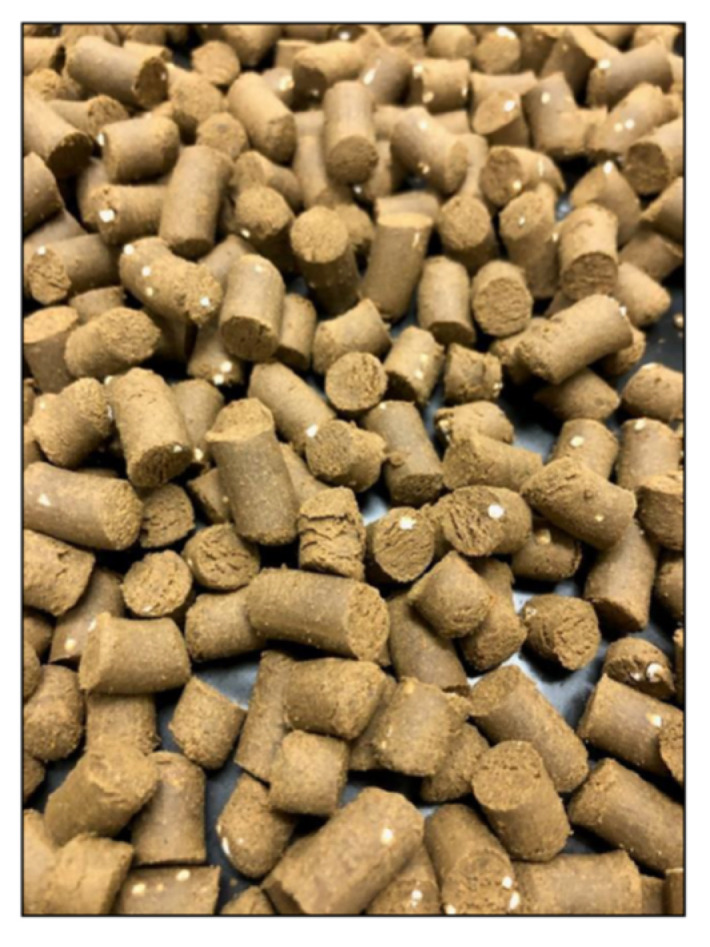
Fish feed incorporated with the PZQ-loaded beads.

Beads of each formulation were incorporated into fish diet pellets by mixing the dry beads with ground commercial yellowtail kingfish feed (Pelagica, Ridley Agriproducts) and extruding the mix into 3 mm or 9 mm diameter pellets using an Imperia and Monferrina Dolly pasta maker (Moncalieri, Italy) (Figure 10). An unmedicated control diet, containing the ground commercial yellowtail kingfish feed only (no PZQ), was made using the same process. All PZQ diets contained the equivalent of 10 g of PZQ/kg fish feed.

Palatability trials were conducted in unreplicated 1.5 m^3^ tanks, unless stated otherwise. Ambient temperature seawater was supplied to all tanks at circa 8 L/min, with a central airstone providing aeration to the tanks. Yellowtail kingfish (without parasites) were fed each treatment diet at a daily fixed ration, calculated using the method of Masumoto [53], which is based on fish body weight and rearing water temperature. The ration was offered for a maximum of 3 min in a single morning feed. If the fish consumed the entire feed ration within 3 min, the time was recorded. After 3 min, any uneaten or regurgitated pellets in each tank were collected using a net attached to the outflow pipe, and the pellets were individually counted. The average dry weight of individual pellets from each diet was calculated and used to determine the equivalent dry weight of uneaten wet feed collected from each tank after each feed. Any remaining dry feed not consumed within the 3 min was also weighed. Both weights were then combined to find the percentage of the feed ration consumed daily in each tank. The data were analysed with the day as the replicates.

Palatability of Formulations A and E was tested over 5 days using either two large yellowtail kingfish (mean body weight 2000 g) or eight small yellowtail kingfish (mean body weight 175 g) per tank. Fish were offered a fixed ration of 48 g and 72 g of food per tank per day in a single morning feed for small and large fish, respectively. Palatability of Formulations B, C, and D was tested over 5 days using ten yellowtail kingfish (mean body weight 260 g) per tank. Fish were offered a fixed ration of 78 g of food per tank per day in a single morning feed.

The digestibility of each formulation was subjectively assessed after the final feed on Day 5. Three fish per medicated treatment were euthanised, by anaesthesia (Isoeugenol, AQUI-S^®^ New Zealand Ltd., Lower Hutt, New Zealand, 40 mg/L), followed by cutting the cervical spine and dissection 3 h post feeding to determine if there were undigested beads remaining in the digestive tract.

### 4.5. Palatability and Efficacy Assessments in Parasite-Infected Fish

A palatability and efficacy trial was conducted on fish infected with gill parasites (*Zeuxapta seriolae*). Two treatment diets incorporating Formulation B and Formulation C were tested against positive (pure PZQ) and negative (unmedicated) control diets. Pellets were prepared as described above, and medicated pellets contained the equivalent of 10 g PZQ/kg feed.

Twelve 4.5 m^3^ tanks were used for the evaluation of the treatments in triplicate. Each tank was stocked with eight yellowtail kingfish (mean weight 1600 g). The fish were acclimated to the experimental system for a period of six days, during which time they were fed to satiety once daily on an unmedicated control diet. The average food intake during this acclimation period was used to calculate the fixed ration of the experimental diets offered. The fish in each tank were offered a fixed ration of 155 g of the treatment diet in a single morning feed over a 6-day period.

The percentage of the ration consumed, and the time taken to do so was recorded as previously described. Based on the actual amount of diet consumed and the PZQ dietary inclusion level (10 g/kg), the PZQ dose received by the fish in each tank (mg/kg) was calculated.

On Day 6, the fish in each tank were anaesthetised (AQUI-S^®^, 20 mg/L) and bathed in PZQ (50 mg/L) for 10 min to assess gill parasite abundance. The PZQ bath water was filtered to 100 µm and the filtrate fixed in a 10% formalin solution. The number of gill parasites in the fixed solution were counted and the percentage reduction in each of the medicated treatment groups was calculated relative to the unmedicated control treatment. Fish fed the Formulation B and Formulation C medicated diets were then euthanised and dissected 3 h post feeding to determine if there were undigested beads remaining in the digestive tract.

### 4.6. Statistical Analysis

For the palatability trial comparing small and large yellowtail kingfish, the data were analysed by two-way ANOVA to determine the effect of fish size and bead type on feed consumption and time taken to consume an entire ration. For all other trials, the results were analysed by one-way ANOVA to determine the effect of bead type on feed consumption, time taken to consume an entire ration, and gill parasite abundance. Data were normalised by arcsine transformation if needed. Significance was accepted at *p <* 0.05. The data were analysed using JMP software (Version 14, SA Institute Inc., Montreal, QC, Canada).

## 5. Patents

Fish Feed Additives, Patent No. 2020901814, 2 June 2020. Inventors: Tang, E., Partridge, G. and Lim, L.Y.

## Figures and Tables

**Table 1:** Drug loading and diameter of dry beads of different praziquantel (PZQ) formulations. Formulations A and E were prepared as 10 g-batches. Formulations B, C, and D were prepared as 10 g and 100 g-batches, and bead diameter was determined from 10-g batches. Data represent mean ± SD.

Formulation (Polymer Matrix)	Drug Loading (% *w*/*w*, *n* = 3)		Diameter of Dry Beads (mm, *n* = 20)
	10 g-Batch	100 g-Batch	
A (chitosan complexed for 3 h)	82.5 ± 0.1	N/A	1.89 ± 0.26
B (alginate and chitosan)	74.7 ± 3.4	78.2 ± 0.3	3.03 ± 0.35
C (agar)	77.8 ± 0.1	80.2 ± 1.5	1.80 ± 0.26
D (alginate and Cremophor^®^ RH40)	83.9 ± 1.3	86.1 ± 0.4	1.36 ± 0.29
E (chitosan complexed overnight)	81.5 ± 1.1	N/A	1.86 ± 0.28
